# Supplementary material for: Developmental variability channels mouse molar evolution
Source: eLife. 2020 Feb 12;9:e50103. doi: 10.7554/eLife.50103 (PMC7182435; doi:10.7554/eLife.50103)
Supplement: Supplementary file 1. [file elife-50103-supp1.docx]

**Supplementary File 1: Scoring criteria used to assess embryonic dental epithelia**

| Criterion | Severity | Phenotype |
| --- | --- | --- |
| R2 *Shh* expression | 0 | no expression |
|  | 1 | weak expression in a small zone |
|  | 2 | strong expression in a larger zone |
| M1 *Shh* expression | 0 | no expression |
|  | 1 | early signaling center: strong expression in a circular zone |
|  | 2 | mature signaling center: strong expression in a large oval zone |
| Cap transition | 0 | width constant along length of dental epithelium |
|  | 1 | slight widening of dental epithelium near the signaling center |
|  | 2 | cap transition completed |
| Anterior protrusion | 0 | dental epithelium flat in R2 zone |
|  | 1 | conspicuous protuberance in R2 zone |
