# Supplementary material for: Developmental variability channels mouse molar evolution
Source: eLife. 2020 Feb 12;9:e50103. doi: 10.7554/eLife.50103 (PMC7182435; doi:10.7554/eLife.50103)
Supplement: Supplementary file 3. [file elife-50103-supp3.docx]

**Supplementary File 3: summary of lineage tracing experiments**

Note: All specimens after tamoxifen administration at 12.5, 13.5, 14.5 dpc respectively were harvested at 17.5 dpc.

| Tamoxifen administration | Injection order | Number of positive specimens in the distinct Cre activation | Method of visualization |
| --- | --- | --- | --- |
| 12.5 | 1. | 4 | Whole mount X-gal staining |
|  | 2. | 1 | Dissociation of epithelia |
|  | 3. | 3 | Dissociation of epithelia |
|  | 4. | 2 | Dissociation of epithelia |
|  | TOTAL | 10 |  |
| 13.5 | 1. | 2 | Whole mount X-gal staining |
|  | 2. | 3 | Whole mount X-gal staining |
|  | 3. | 2 | Dissociation of epithelia |
|  | 4. | 1 | Dissociation of epithelia |
|  | 5. | 2 | Dissociation of epithelia |
|  | TOTAL | 10 |  |
| 14.5 | 1. | 2 | Whole mount X-gal staining |
|  | 2. | 1 | Dissociation of epithelia |
|  | 3. | 2 | Dissociation of epithelia |
|  | 4. | 4 | Dissociation of epithelia |
|  | TOTAL | 9 |  |
