# Supplementary material for: Developmental variability channels mouse molar evolution
Source: eLife. 2020 Feb 12;9:e50103. doi: 10.7554/eLife.50103 (PMC7182435; doi:10.7554/eLife.50103)
Supplement: Supplementary file 4. [file elife-50103-supp4.docx]

**Supplementary statistical details**

The following tables provide summaries of key statistical tests performed for this study.

**Comparing the timing of developmental events between strains**

Logistic regression

**Comparisons of dental lamina measurements**

t-test

**Comparison of outcomes of culture between strains**

Fisher’s exact test

**Comparisons of proportions of up/downregulated genes**

Chi-squared tests

**Comparing rarity of R2 spot between upper and lower jaws**

Cross-tabulation (lower and upper sample from the same embryo)

Fisher’s exact test

**Overall variability of developmental trajectories**

Wilcoxon rank-sum test

**Comparisons of variation in adult molar length/width**

test for equality of two variances between two groups, performed in Systat.

| **comparison** | **jaw** | **n** | **p-value** |
| --- | --- | --- | --- |
| DUHi/FVB length variation | Upper | 30 | 0.095 |
| DUHi/FVB width variation | Upper | 30 | 0.996 |
| DUHi/FVB length variation | Lower | 30 | 0.710 |
| DUHi/FVB width variation | Lower | 30 | 0.111 |
