# Supplementary material for: Developmental variability channels mouse molar evolution
Source: eLife. 2020 Feb 12;9:e50103. doi: 10.7554/eLife.50103 (PMC7182435; doi:10.7554/eLife.50103)
Supplement: Transparent reporting form [file elife-50103-transrepform.pdf]

1st Floor
24 Hills Road
Cambridge CB2 1JP, UK

P 01223 855340
W elifesciences.org
T @elife

eLife's transparent reporting form

We encourage authors to provide detailed information *within their submission* to facilitate the interpretation and replication of experiments. Authors can upload supporting documentation to indicate the use of appropriate reporting guidelines for health-related research (see EQUATOR Network), life science research (see the BioSharing Information Resource), or the ARRIVE guidelines for reporting work involving animal research. Where applicable, authors should refer to any relevant reporting standards documents in this form.

If you have any questions, please consult our Journal Policies and/or contact us: editorial@elifesciences.org.

Sample-size estimation

- You should state whether an appropriate sample size was computed when the study was being designed
- You should state the statistical method of sample size computation and any required assumptions
- If no explicit power analysis was used, you should describe how you decided what sample (replicate) size (number) to use

Please outline where this information can be found within the submission (e.g., sections or figure legends), or explain why this information doesn't apply to your submission:

eLife Sciences Publications, Ltd is a limited liability non-profit non-stock corporation incorporated in the State of Delaware, USA, with company number 5030732, and is registered in the UK with company number FC030576 and branch number BR015634 at the address 1st Floor, 24 Hills Road, Cambridge CB2 1JP | August 2014

Morphometrics (Figure 1 and 6, supplementary figure 10)
Sample size per species was determined by sample availability in collections (lowest sample size: n=4).

DUHi/FVB comparison (Figures 2, 3, 4, 6 and Supplementary Figures 2-8, 10)
The concept of replicates is inherently problematic in a developmental context, as the dynamic nature of development makes it difficult to define one embryo as a replicate of another.
Due to the chronic lack of quantitative data on variation in developmental dynamics in mammalian systems (an issue which this study tries to address), we were unable to make reasonable assumptions as to the expected effect sizes of the type of effects we hoped to uncover. This precluded explicit power analyses and also meant that we needed to take particular care to ensure the depth and breadth of our sampling approach.
In this study each embryo was given a numeric age according to its weight or its computed age (based on weight and day of copulation), then used to define class of embryos that have or do not have the same phenotype. As opposed to many studies harvesting discontinuous timepoints, we used several independent litters, continuously covering the whole developmental period of investigation. We did our best to deal with the natural variability in fertilization and development to adjust as most as realistically possible sampling size along developmental time. Sampling can be directly visualized in many figures (figure 3, sup fig 5-8; supplementary methods).

Lineage tracing (Figure 5):
Explicit power analysis was not used because the lineage tracing experiment follows an all-or-nothing principle, in the sense of presence or absence of labelled cells. Obtaining embryos is made difficult by the necessity to combine 2 genetic modifications, therefore we stopped experiments after having obtained at least three independent experiments (injections) with variable number of embryos with the right genotype, all showing the same result (presence or absence of staining in the anterior part of the lower or upper molar germ).

Transcriptomics (Figure 4 and Supplementary figure 9)
RNAseq experiment was performed in three 3 replicates per condition (jaw and strain), a standard in RNAseq analysis with DE2seq.

Replicates

- You should report how often each experiment was performed
- You should include a definition of biological versus technical replication
- The data obtained should be provided and sufficient information should be provided to indicate the number of independent biological and/or technical replicates
- If you encountered any outliers, you should describe how these were handled
- Criteria for exclusion/inclusion of data should be clearly stated
- High-throughput sequence data should be uploaded before submission, with a private link for reviewers provided (these are available from both GEO and ArrayExpress)

Please outline where this information can be found within the submission (e.g., sections or figure legends), or explain why this information doesn't apply to your submission:

Morphometrics (Figure 1 and 6, supplementary figure 10)
For all strains and species, one first upper molar (either right or left) was measured for all available specimens, to the only exclusion of specimens for which the molar was damaged on both side. The outline was measured manually; the amount of measurement error was controlled based on previous studies on similar material (see Renaud et al. *Evolution and Development* 2009). The impact of measurement error was limited by choosing an adequate threshold in the series of harmonics.

DUHi/FVB comparison (Figures 2, 3, 4, 6 and Supplementary Figures 2-8, 10)
See above section sample size estimation for a discussion about replicates in this study. All embryos sampled (i.e. excluding embryos obviously malformed or with severe developmental delays) were used in the study.

Transcriptomics (Figure 4 and Supplementary figure 9)
Due to the dynamic nature of development, there is no true "replicate" in developmental biology: only replicates that are more or less at the same developmental stage. Due to the rapid rhythm of tooth development in mouse and the lack of high-resolution morphological signs, it is impossible to pre-select samples at exactly equivalent stages. The best we can do is to reveal stages differences *a posteriori* from the transcriptome. Therefore, we took our 3 replicates from a single litter, ordering them in time from their fine-scale differences in body weight. This reveals the short-time dynamics of the transcriptome and enables us to control *a posteriori* for stages differences, when studying the transcriptomes Our approach is detailed in the supplementary text 1. Data are available in Supplementary File 1 and on a github: https://github.com/msemon/trDUHi_FVB.
Dissections were performed by a single highly experimented manipulator. From each embryo, we prepared RNA by pooling left and right molar germ, what averages dissection issues.

Lineage tracing (Figure 5):
Each of the 3 different lineage tracing experiments was replicated 4 to 5 times (i.e. 4 to 5 independent injections, each giving raise to 1 to 4 embryos with the right genotype). Patterns of labeled cells could vary as a result of developmental stages differences within a litter or between litters at injection or harvesting, but all embryos (n=10) injected at 12.5 dpc were consistently showed anterior labelling in upper M1 germ that was absent in 13.5 and 14.5 dpc injected embryos (n=10 and n=9). See Supplementary Table 2.

eLife Sciences Publications, Ltd is a limited liability non-profit non-stock corporation incorporated in the State of Delaware, USA, with company number 5030732, and is registered in the UK with company number FC030576 and branch number BR015634 at the address 1st Floor, 24 Hills Road, Cambridge CB2 1JP | August 2014

Statistical reporting

- Statistical analysis methods should be described and justified
- Raw data should be presented in figures whenever informative to do so (typically when N per group is less than 10)
- For each experiment, you should identify the statistical tests used, exact values of N, definitions of center, methods of multiple test correction, and dispersion and precision measures (e.g., mean, median, SD, SEM, confidence intervals; and, for the major substantive results, a measure of effect size (e.g., Pearson's r, Cohen's d)
- Report exact p-values wherever possible alongside the summary statistics and 95% confidence intervals. These should be reported for all key questions and not only when the p-value is less than 0.05.

Please outline where this information can be found within the submission (e.g., sections or figure legends), or explain why this information doesn't apply to your submission:

Morphometrics (Figure 6, supplementary figure 10)
A summary of Systat test results is provided in the supplementary statistical details section.

DUHi/FVB developmental state comparison (Figures 2, 3, 4, 6 and Supplementary Figures 2-8, 10)
Statistical methods used are described in detail in the materials & methods section, with particular attention paid to novel methods of measuring developmental variation. Codes to perform the tests are provided on a github: https://github.com/luke-hayden/dvpap/devstate.
The results of statistical tests, along with sample sizes in each case, are summarized in the supplementary statistical details section.

DUHi-FVB developing morphology comparison (Figure 4, Supplementary Figure 7)
The results of statistical tests, along with sample sizes in each case, are summarized in the supplementary statistical details section.

Transcriptomics (Figure 4 and Supplementary figure 9)
The tests performed are classically used in transcriptomics. We are providing the corresponding R code with specific DESeq models. Adjusted p-values as computed by DEseq2 are provided in Supplementary File 1. A threshold of padj<0.05 was used as recommended in the package.
The list of BMP4 and Wnt targets activated and repressed target genes was established by reviewing a list of regulatory relationships published by O'Connell et al. 2012 (i.e. positive or negative effect on gene expression level upon up- or down-regulation of the pathway in different genetic conditions or following pharmacological treatments). These original data including references for the regulatory relationship and our classification as an activated or repressed target of the pathway are listed in supplementary file 1. A summary of Chi-square test results for these data is provided in the supplementary statistical details section.

(For large datasets, or papers with a very large number of statistical tests, you may upload a single table file with tests, Ns, etc., with reference to sections in the manuscript.)

Group allocation

- Indicate how samples were allocated into experimental groups (in the case of clinical studies, please specify allocation to treatment method); if randomization was used, please also state if restricted randomization was applied
- Indicate if masking was used during group allocation, data collection and/or data analysis

Please outline where this information can be found within the submission (e.g., sections or figure legends), or explain why this information doesn't apply to your submission:

Not applicable.

Additional data files ("source data")

- We encourage you to upload relevant additional data files, such as numerical data that are represented as a graph in a figure, or as a summary table
- Where provided, these should be in the most useful format, and they can be uploaded as "Source data" files linked to a main figure or table
- Include model definition files including the full list of parameters used
- Include code used for data analysis (e.g., R, MatLab)
- Avoid stating that data files are "available upon request"

Please indicate the figures or tables for which source data files have been provided:

Bayesian model of embryonic age estimation (Figures 3, 4, 6 and Supplementary Figure 5-8, 10)
The initial data, R source code, parameters and raw results are available on github: https://github.com/msemon/cdpc

DUHi/FVB developmental state comparison (Figures 2, 3, 4, 6 and Supplementary Figures 2-8, 10)
Two tables with all analyzed embryos, with their weight, age in cdpc, information on litter and scored characteristics, and the code used to analyze these data is provided on github: https://github.com/luke-hayden/dvpap/devstate

DUHi-FVB developing morphology comparison (Figure 4, Supplementary Figure 7)
A table with all embryo measurements, weights and other relevant characteristics used to analyse these data is provided on github: https://github.com/luke-hayden/dvpap/devmorph

Transcriptomics (Figure 4 and Supplementary figure 9)
Processed data with statistics are provided in Supplementary File 1. Processed and raw data were desposited on GEO, accession number GSE135432. The mapping data (by Kallisto, on each reference strain as discussed in the text), R source code and parameters are available on github https://github.com/msemon/trDUHi_FVB. Code used for graphing is provided here: https://github.com/luke-hayden/dvpap/transcriptofig

Lineage tracing (Figure 5):
A table with all analyzed embryos from different injection conditions is provided as Supplementary Table 2.

Morphometrics (Figure 1 and 6)
A table with fourrier coefficients, measures of Length and width is available : https://github.com/luke-hayden/dvpap/devmorph

eLife Sciences Publications, Ltd is a limited liability non-profit non-stock corporation incorporated in the State of Delaware, USA, with company number 5030732, and is registered in the UK with company number FC030576 and branch number BR015634 at the address 1st Floor, 24 Hills Road, Cambridge CB2 1JP | August 2014
